# Phenotypic characterization of Nguni goats in four agro-ecological zones of Limpopo province, South Africa

**DOI:** 10.1371/journal.pone.0314408

**Published:** 2024-12-04

**Authors:** Madumetja Cyril Mathapo, Joseph Thinawanga Mugwabana, Thobela Louis Tyasi

**Affiliations:** School of Agriculture and Environmental Sciences, Department of Agricultural Economics and Animal Production, University of Limpopo, Polokwane, Sovenga, Limpopo, South Africa; Universidade Federal de Mato Grosso do Sul, BRAZIL

## Abstract

The study was conducted to phenotypically characterize Nguni goats from four agro-ecological zones of Limpopo province, South Africa. A total of 426 goats were sampled from four agro-ecological zones. The quantitative traits that were studied for phenotypic characterization using Analysis of Variance (ANOVA) were heart girth (HG), body length (BL), withers height (WH), sternum height (SH), rump height (RH), rump length (RL), rump width (RW), cannon circumference (CC), testicular length (TL) and scrotal circumference (SC) while qualitative traits were coat colour pattern and type, back profile, ear orientation, presence of horn, horn shape and orientation. The study further looked at the structural indices of the goats. The findings indicated that the agro-ecological zones significantly affected (P<0.05) the quantitative traits of Nguni goats. Female goats from arid zone had higher (P<0.05) BW, HG, BL, WH, RH, and CC with mean values of 35.76±0.92, 73.89±0.63, 66.26±0.62, 63.40±0.56, 64.71±0.44 and 8.07±0.06, respectively as compared to the other zones. Male goats from arid zone had higher (P<0.05) BW, HG, BL, RW, TL and SC with mean values of 37.20±2.29, 78.75±1.44, 70.95±1.57, 16.83±0.38, 15.50±0.50 and 31.00±0.00, respectively as compared to other zones. In terms of qualitative traits, the goats were characterized by higher proportion of patchy coat colour pattern (62.7%), brown dominant colour type (42.6%), straight back profile (46.6%), and semi-pendulous ear orientation (65.4%). The goats had horns (100%), curved horn shape (71.6%) and backward horn orientation (89.4%). Though the agro-ecological zone had non-significant influence (P>0.05) on the qualitative traits. Findings of structural indices on dactyl-thoracic, transversal pelvic, longitudinal pelvic and proportionality indicated that the Nguni goats can be matched as light medium meat type. In conclusion, the variation in morphometric traits of Nguni goats and knowing their structural and functional indices can assist in their conservation and genetic improvement. There was no difference in the qualitative traits of the goats in four agro-ecological zones. Studies needed to be conducted on genetic characterization using genetic markers to integrate the information from morphological traits.

## Introduction

Goats farming is an industry that has been practised by a large group of people at a rural level due to the crucial role they play to their lives [1]. Indigenous goats provide farmers with limited resources food, economic security, and advantages to their customs and culture [2]. Indigenous goats are breeds kept under extensive production and can be able to withstand severe environmental circumstances while still contributing towards food security [3]. Despite their contribution, there is genetic erosion of indigenous goats in developing countries, which expose them to the danger of extinction [4]. This is caused by the crossbreeding with exotic breeds that reduces the adaptive value of this indigenous goats [5]. One of the challenges in conservation of livestock genetic resources is lack of knowledge on the best ways for animal characterization and production systems [6]. The morphological characterization of native livestock genetic resources is fundamental for classification of any livestock resources, the classification is focusing on their size and shape which can be serve as economic indicators for why there are reared [2]. Several studies [6–8] have been undertaken on morphological characterization of indigenous goats for conservation of their genetic resources and showed variation, where some goats displayed, small, medium and large body frame. However, according to our knowledge there is a limited information on the effect of agro-ecological zones on morphological characterization and structural indices of Nguni goats. The structural indices, which describe the kind and function of the animal, are expressed as percentages and are the results of combining two or more morphometric measures [9]. Thus, the purpose of this study was to assess the effect of agro-ecological zones on morphological characterization and structural indices of Nguni goats in Limpopo province, South Africa. This study will be useful to researchers when developing a breeding program that will preserve and enhance the genetic makeup of Nguni goats. It will also benefit the smallholder farmers to improve the productivity of their goats by being able to select best animals for breeding.

## Materials and methods

### Ethical approval

Before the start of the study, ethical approval was given by the University of Limpopo Animal Research Ethics Committee (ULAREC) under the number: AREC/44/2023: PG.

### Study area

The study was carried out in four districts of the Limpopo Province, South Africa, which represents distinct agro-ecological zones. The agro-ecological zone varies according to climatic conditions, altitudes, soil types and vegetation type [10]. The arid zone has the sweet and mixed veld with a rainfall of less than 254mm, and 15 to 38°C. Semi-arid zone has the sour and mixed veld with rainfall between 254 and 508 mm, and 25 to 35°C. Humid zone has sour veld with rainfall between 1500 to 2500mm, and temperature of 24 to 27°C. Sub-humid has a sweet veld with a rainfall Less than 1500mm, and 27 to 35°C temperatures [10].

### Experimental animals and management

Nguni goats between the ages of one and four years old were used in the study. The focus of the study was goats that were used for breeding purposes. In the morning, they were free to graze and browse, and in the afternoon, they were closed in the paddocks. Animals received seasonal vaccinations as well as routine tick-exclusion dips. In the trial, sick and pregnant animals were eliminated.

### Study design

A cross-sectional study design was employed in the study where data was collected from each individual animal once [11]. Therefore, farmers from each agro-ecological zones were visited during data collection, where the animals were measured, and farmers interviewed once.

### Sample size

The study used goats of the Nguni goat farmers registered under the Indigenous Veld Goats (IVGs) association in Limpopo Province that was established in 2015 by Mathuba Genetics. A sample size of 369 goats was appropriate for assessing both qualitative and quantitative features in this kind of research, according to Mdladla et al. [12]. As a results, 426 goats as the sample of this study were larger than the sample size advised by Mdladla et al. [12]. A total of 30 farmers out of 85 Nguni goat farmers in the province were randomly selected for data collection on their Nguni goats. Whannou et al. [7] suggests that a sample size of 5 to 20 farmers, depending on their interest in taking part in this type of study, is suitable.

### Sampling procedure

The study used purposive sampling where four districts (Vhembe, Capricorn, Mopani, and Sekhukhune) representing different agro-ecological zones (Arid, Semi-arid, Humid, and Sub-humid) within Limpopo province were selected due to presence of IVGs Nguni goat farmers. A total of 30 Nguni goat farmers out of 85 farmers from four districts of Limpopo were randomly selected based on their interest to participate in the study. The study used a total of 426 Nguni goats in Limpopo Province for quantitative traits.

### Data collection

Together with a visual evaluation of the goat’s appearance and measurements made using an FAO standard format modified from breed descriptor list [13], the qualitative data (coat colour type, coat colour pattern, horn presence, horn shape, horn orientation, and ear orientation) were recorded (S1 File). Quantitative traits studied (S2 File) were body weight (BW) which was measured using a weighing scale calibrated in kilograms (kg) and the morphometric traits such as heart girth (HG), body length (BL), withers height (WH), rump height (RH), rump length (RL), rump width (RW), sternum height (SH), cannon circumference (CC), testicular length (TL) and scrotal circumference (SC) were taken using tailor measuring tape calibrated in centimetres (cm). The measurements were taken early in the morning before the animals were released for grazing. The age of the animals was estimated using dentition scale. Study used non-pregnant and healthy animals during data collection.

### Structural indices

Four conformation indices were formulated according to Barragan [14] as follows: Dactyl-thoracic (DTI): this method gives an indication of the degree of skeletal fineness by categorising the animals into three groups: hypermetric (big format), eumetric (middle format), and orelipometric (small format). Animals with a dairy phenotype are classified as less than 10, while those with a meat phenotype are classified as greater than 11. The measurements of the animal’s meat aptitude, known as the transversal pelvic index (TPI) and longitudinal pelvic index (LPI), are based on the length and width of the rump in relation to the height at withers, respectively. A TPI significantly higher than 33 and an LPI no higher than 37 are appropriate markers of meat animals. The relative thickness of cannon bone (RTI), which is larger in breeds with a high meat content, illustrates the relationship between the animal’s height and the circumference of the cannon bone. Proportionality (PRI), which indicates an animal’s shape, connects body height to body length. A value below 100 indicates that the animal’s form tends to be rectangular, which is typical of meat breeds; a value above 100 suggests that the animal’s form tends to be square, which is typical of dairy breeds. Table 1 contains the procedures for computing body indices and their types.

**Table 1:** Structural indices and their formulas.

Structural indices	Formulas
Dactyl-thoracic (DTI)	$DTI=\frac{Cannon\;bone}{heart\;girth}\times 100$
Transversal pelvic index (TPI)	$TPI=\frac{rump\;width}{withers\;height}\times 100$
Longitudinal pelvic index (LPI)	$LPI=\frac{rump\;length}{withers\;height}\times 100$
Relative thickness of cannon bone (RTI)	$RTI=\frac{cannon\;bone}{withers\;height}\times 100$
Proportionality (PRI)	$PRI=\frac{withers\;height}{body\;lenth}\times 100$

Source: Barragan [14].

### Statistical analysis

Statistical Package for Social Sciences [15] version 29.0 was used for data analysis. To obtain the qualitative results, descriptive statistics like percentages and frequencies as well as Chi-square test were employed, to check significant difference (P<0.05) among the agro-ecological zones. The Analysis of Variance (ANOVA) was used to determine effect of agro-ecological zones on morphometric traits of goats. The Fisher’s Least Significant Difference (LSD) test was used to test significant difference (P<0.05) of least-square means. Below is the General Linear model that was used:

$$y_{ij}=u+A_{i}+e_{ij}$$

Where:

*Yij* = the observation on body weight and morphometric traits;

*μ* = overall mean;

*A*i = fixed effect of agro-ecological zones;

eij = random error

## Results

Table 2 shows the effect of agro-ecological zones on morphometric traits of female and male Nguni goats. The findings indicated that agro-ecological zones had a significant effect (P<0.05) on all morphometric traits of female goats. The female goats from arid zone showed high mean values of BW, HG, BL, RH, and CC as compared to female goats from other agro-ecological zones. Female goats from arid zone had similar WH mean value with goats from semi-arid but different with those from humid and sub-humid. Female goats from arid zone had similar RL mean value with goats from semi-arid, semi-arid and humid zones. Agro-ecological had a significant effect (P<0.05) on some morphometric traits of male goats such as BW, HG, BL, RL, RW, SH, TL and SC, and non-significant effect (P>0.05) on WH, RH and CC. Male goats from arid zone had a high mean value of BW as compared to goats from sub-humid but similar to those from semi-arid and humid zones. Findings indicated that male goats from arid zone had high mean value of HG, BL, RW, TL and SC as compared to males from other agro-ecological zones.

**Table 2:** Morphometric traits of Nguni goats in different agro-ecological of Limpopo province.

Aro-ecological zones										
Variables	Arid		Semi-arid		Humid		Sub-humid		Overall	
	Female	Male	Female	Male	Female	Male	Female	Male	Female	Male
	N (82) Mean±SE	N (24) Mean±SE	N (81) Mean±SE	N (12) Mean±SE	N (97) Mean±SE	N (10) Mean±SE	N (97) Mean±SE	N (23) Mean±SE	N (357) Mean	N (69) Mean
BW	35.76±0.92^a^	37.20±2.29^a^	32.48±0.80^b^	34.08±2.55^ba^	32.05±0.47^cb^	32.00±4.02^ba^	30.21±0.75^c^	28.60±1.73^b^	32.63	32.97
HG	73.89±0.63^a^	78.75±1.44^a^	71.83±0.76^b^	71.83±1.97^b^	71.64±0.42^b^	70.10±3.86^b^	68.94±0.61^c^	67.43±1.68^b^	71.58	72.03
BL	66.26±0.62^a^	70.95±1.57^a^	65.41±0.68^a^	64.33±1.72^b^	63.65±0.46^b^	62.10±3.00^b^	63.17±0.60^b^	63.21±1.71^b^	64.62	65.15
WH	63.40±0.56^a^	65.91±1.56	62.95±0.50^a^	61.50±1.28	60.02±0.40^b^	62.40±1.96	60.04±0.45^b^	61.08±1.23	61.60	62.72
RH	64.71±0.44^a^	63.45±1.65	62.35±0.43^b^	62.66±1.18	60.06±0.43^c^	63.20±2.23	63.70±0.49^a^	62.26±1.62	62.71	62.89
RL	20.41±0.32^a^	16.66±0.98^b^	20.48±0.23^a^	20.00±0.66^a^	20.27±0.12^a^	19.70±1.02^a^	18.80±0.19^b^	19.39±0.44^a^	19.99	18.94
RW	16.23±0.22^a^	16.83±0.38^a^	16.18±0.76^a^	13.66±0.41^b^	14.48±0.17^b^	14.20±0.78^ba^	15.03±0.20^b^	14.60±1.25^ba^	15.48	14.82
SH	42.01±0.59^b^	39.25±1.30^b^	45.02±0.53^a^	42.75±1.74^ba^	42.21±0.36^b^	43.90±1.44^a^	43.17±0.47^b^	44.86±0.93^a^	43.10	42.69
CC	8.07±0.06^a^	8.12±0.23	7.75±0.07^b^	8.16±0.24	7.83±0.07^b^	8.00±0.59	7.61±0.09^b^	8.21±0.25	7.82	8.12
TL	-	15.50±0.50^a^	-	11.66±0.49^b^	-	11.62±0.49^b^	-	12.13±0.35^b^	-	12.73
SC	-	31.00±0.00^a^	-	21.83±0.90^b^	-	22.12±0.69^b^	-	26.20±1.72^ba^	-	25.29

SE: Standard Error; BW: Body weight; HG: Heart girth; BL: Body length; WH: Withers height; RH: Rump height; RL: Rump length; RW: Rump width; SH: Sternum height; TL: Testicular length; SC: Scrotal circumference; P-Value: Significance level.

Table 3 indicates the structural indices for Nguni goats from four agro-ecological zones of Limpopo province, South Africa. Goats had similar dactyl-thoracic values in all agro-ecological zones. The goats from arid zones had high transversal pelvic, followed by those from semi-humid, semi-arid and humid. Goats from humid zone showed high longitudinal pelvic, followed by semi-arid and semi-humid and arid zones. Goats from semi-humid, humid, and arid had high similar relative thickness of cannon bone, then followed by semi-arid zone.

**Table 3:** Calculated structural and functional indices from morphometric traits of Nguni goats in four agro-ecological zones of Limpopo province.

Agro-ecological zones					
Body indices	Semi- humid (%)	Humid (%)	Semi- arid (%)	Arid (%)	Overall (%)
Dactyl-thoracic (DTI)	11	11	11	11	11
Transversal pelvic (TPI)	25	24	25	26	25
Longitudinal pelvic (LPI)	31	34	33	31	32
Relative thickness of cannon bone (RTI)	13	13	12	13	13
Proportionality (PRI)	94	96	96	95	95

%: Percentages

**Fig 1:**
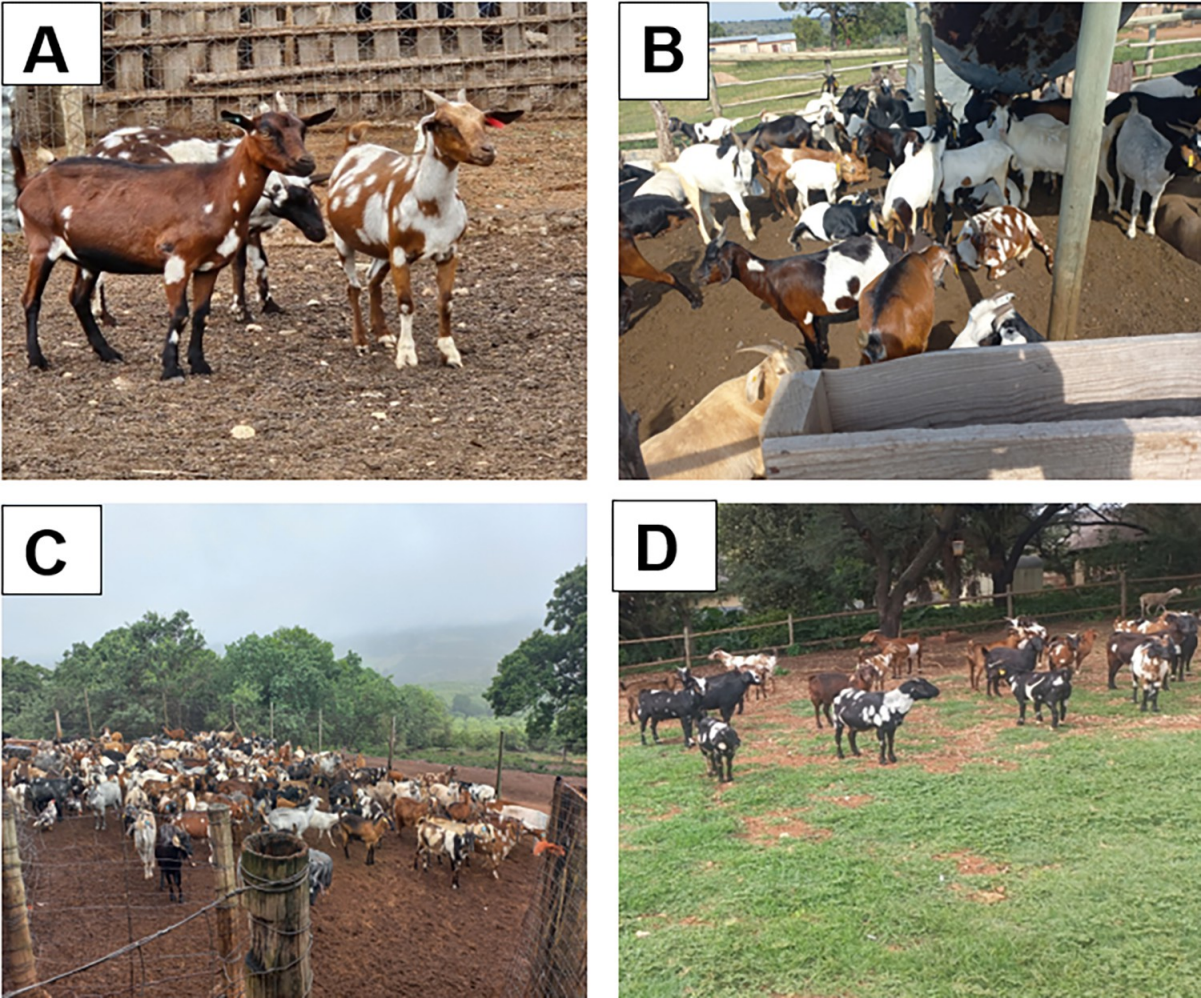
Shows coat colour type and pattern from different agro-ecological zones. A: Sub-humid; B: Arid; C: Humid; D: Semi-arid (Photographs by MC Mathapo).

The Nguni goats found in the semi-arid zone (Table 4) mostly had patchy coat colour pattern (85.7%), followed by the spotted (14.3%) while in the semi-humid zone the dominating pattern was plain (40%) followed by patchy and spotted with (30%), respectively. In humid zone, the dominating coat colour pattern was patchy (60%), followed by spotted (30%) while in arid zone, patchy was dominant (75%), followed by plain (12.5%) and spotted (12.5%). The findings further showed non-significant difference (P>0.05) on coat colour patterns (Fig 1) between the agro-ecological zones.

**Table 4:** Qualitative traits of Nguni goats found in Limpopo province.

Argo-ecological zones						
Traits	Attributes	Semi-Arid N (%)	Semi-Humid N (%)	Humid N (%)	Arid N (%)	Overall N (%)
Coat	Plain	0 (0.0%)	4 (40%)	0 (0.0%)	1 (12.5%)	5 (13.1%)
Color	Patchy	6 (85.7%)	3 (30%)	3 (60%)	6 (75%)	18 (62.7%)
pattern	Spotted	1 (14.3%)	3 (30%)	2 (40%)	1 (12.5%)	7 (24.2%)
*x* ^2^						0.16
Coat	White	0 (0.0%)	2 (20%)	0 (0.0%)	0 (0.0%)	2 (5%)
Type	Black	0 (0.0%)	1 (10%)	1 (20%)	1 (12.5%)	3 (10.6%)
	Brown	0 (0.0%)	0 (0.0%)	0 (0.0%)	1 (12.5%)	1 (3.1%)
	White dominant	4 (57.1%)	2 (20%)	1 (20%)	2 (25%)	9 (30.5%)
	Black dominant	0 (0.0%)	2 (20%)	0 (0.0%)	1 (12.5%)	3 (8.1%)
	Brown dominant	3 (42.9%)	3 (30%)	3 (60%)	3 (37.5%)	12 (42.6)
*x* ^2^						0.58
Back profile	Slope up the rump	2 (28.6%)	2 (20%)	3 (60%)	1 (12.5%)	8 (30.7%)
	Straight	5 (71.4%)	4 (40%)	0 (0.0%)	6 (75%)	15 (46.6%)
	Slope down the withers	0 (0.0%)	0 (0.0%)	1 (20%)	0 (0.0%)	0 (0%)
	curved	0 (0.0%)	4 (40%)	1 (20%)	1 (12.5%)	6 (18.1%)
*x* ^2^						0.07
Ear orientation	Erect	2 (28.6%)	5 (50%)	0 (0.0%)	3 (37.5%)	10 (29%)
	Semi pendulous	5 (71.4%)	4 (40%)	5 (100%)	4 (50%)	18 (65.4%)
	Pendulous	0 (0.0%)	1 (10%)	0 (0.0%)	0 (0.0%)	1 (2.5%)
	Carried horizontal	0 (0.0%)	0 (0.0%)	0 (0.0%)	1 (12.5%)	1 (3.1%)
*x* ^2^						0.38
Horns	Present	7 (100%)	10 (100%)	5 (100%)	8 (100%)	30 (100%)
	Absent	-	-	-	-	-
*x* ^2^						
Horn shape	Straight	2 (28.6%)	6 (60%)	0 (0.0%)	2 (25%)	10 (28.4%)
	Curved	5 (71.4%)	4 (40%)	5 (100%)	6 (75%)	20 (71.6%)
*x* ^2^						0.11
Horn orientation	Lateral	0 (0.0%)	1 (10%)	0 (0.0%)	1 (12.5%)	2 (5.6%)
	Obliquely upward	0 (0.0%)	2 (20%)	0 (0.0%)	0 (0.0%)	2 (4%)
	Backward	7 (100%)	7 (70%)	5 (100%)	7 (87.5%)	26 (89.4%)
*x* ^2^						0.43

*x*^2^: Chi-square; N: Number; %: Percentages

On the coat colour type, the most common coat colour among goats in the semi-arid zone was white dominant (57.1%), followed by brown dominant (42.9%), and in the semi-humid zone, brown dominant (30%) was the common coat colour, followed by black dominant (20%), white dominant (20%), and white (20%), and black (10%). Brown dominant colour was the most common coat colour in the humid zone (60%) with the white and black followed with the similar value (20%). The findings further indicated that the most common coat colour type in the arid zone was brown dominant (37.5%), which was followed by white dominant (25%) and black dominant (12.5%), brown and black shared the same value (12.5%). Based on coat colour type (Fig 1), the results indicated that there was no significant difference (P>0.05) across the agro-ecological zones.

The dominant back profile in the semi-arid zone was straight (71.4%), then followed by slope up the rump (28.6%) while in the semi-humid, were curved (40%) and straight (40%), followed by slope up the rump (20%). In the humid zone, the dominant back profile was slope up the rump (60%), followed by slope up the withers (20%) and curved (20%). Then in the arid, was found that the dominating profile was straight (75%), followed by slope up the rump (12.5) and curved (12.5%). The were non-significant difference (P>0.05) on back profiles of the goats from different agro-ecological zones.

With regards to ear orientation, it was found that in semi-arid zone, semi-pendulous (71.4%) dominated, followed by erect (28.6%), whereas in semi-humid zones, erect (50%) dominated, followed by semi-pendulous (40%) and pendulous (10%). All the goats from humid zone had semi-pendulous ears (100%), while in the arid zone semi-pendulous ear orientation dominated and was followed by erect (37.5%) and carried horizontal (12.5%). There was no statistically significant difference (P>0.05) on ear orientation of the goats among the various agro-ecological zones. The results revealed that all goats from all agro-ecological zones had horns, and there was no significant difference (P>0.05) in present of horns between the agro-ecological zones.

Regarding the horn shape, the results indicated that the goats from the semi-arid zone had curved horns (71.4%), which were followed by straight horns (28.6%), while the goats from the semi-humid zone had straight horns (60%), followed by curved (40%). All goats from humid zone had curved (100%) horns, while straight horns dominated in the arid zone (75%). The difference in horn shape between the agro-ecological were not statistically significant (P>0.05). On the horn orientation, all goats from semi-arid zone had backward orientation (100%). Goats from semi-humid zone had backward orientation (70%), followed by obliquely upward (20%) and lateral (10%). All goats from humid zone had backward orientation (100%) while in the arid zone the dominating orientation of horns was backward (87.56%), with lateral coming in second (12.5%). The results showed a non-significant difference (P>0.05) on the horn orientation among the agro-ecological zones.

## Discussion

A deeper comprehension of phenotypic characteristics of animals helps to improve the implementation of conservation strategies aimed at securing the survival of local livestock [16]. The study’s initial focus was on the influence of agro-ecological zones on phenotypic characterization. The current study’s results on female goats showed that all morphometric features were significantly influenced by agro-ecological zones, and that female goats from arid zones had higher body weights, heart girths, body lengths, and cannon circumferences. The current study’s r21esults were consistent with those of Selolo et al. [17], who found that the agro-ecological zones significantly affected all studied morphometric traits of female non-descript indigenous goats of South Africa with mean values of body weight, body length, and heart girth varying across the zones. The current study’s findings, however, were at odds with those of Mohale [18], who concluded that all studied morphometric traits except ear length and chest width, were unaffected by agro-ecological zones. Agro-ecological zones were found to significantly influence several morphological traits in male goats, including body weight, heart girth, body length, rump length, rump breadth, sternum height, testicular attributes, and scrotal circumferences. The results of this study concur with those of Belayhun et al. [19], who found that various morphometric features of male goats were significantly impacted by agro-ecological zones. On the other hand, results of Takele et al. [20], were consistent with those of the male goats and differed with those of the female goats in the current study. The results of the current investigation on male goats and the findings of Yemane et al. [21], showed that morphometric features in all studies were not substantially influenced by agro-ecological zones. Agro-ecological zones’ influence on morphometric features may result from a variety of causes, including animal management practices, the accessibility of local feed resources, and environmental factors [20, 22].

In order to understand how Nguni goats from various agro-ecological zones function, the study also examined structural indices. Dactyl-thoracic index (DTI) measures the fineness of the skeleton and is higher in meat animals than in milk animals [23]. DTI values range from less than 10.5 in light animals to up to 10.8 in intermediate species, 11.0 in light meat animals, and 11.5 in heavy meat animals [14]. Goats belonging to four different agro-ecological zones can be categorised as light meat animals based on the DTI values found in the current study. The current study’s findings were not in line with those of Assefa et al. [24], who claimed that their goats were heavy meat eaters. However, as the DTI values in both investigations were up to 11, the results of Tade et al. [25], on DTI were consistent with the results of the current investigation. According to Silva-Jarquin et al. [26], meat animals should be identified by a longitudinal pelvic index (LPI) no more than 37 and a transverse pelvic index (TPI) significantly greater than 33. The current study’s TPI and LPI results fell short of the declared ranges. The results of this study were consistent with those of Getaneh et al. [27], who found that the TPI and LPI were below both ranges of numbers. According to Assefa et al. [24], the results of the current study on TPI and LPI are indicative of meat-type animals and a medium propensity that permits it to build muscular tissue as well. An animal’s shape is indicated by proportionality (PRI), which links body length and height [14]. When an animal’s value is less than 100, it suggests that its form is more rectangular, which is typical of meat breeds; when it is greater than 100, it suggests that its form is more square, which is typical of dairy breeds [14]. The current study’s PRI results in every agro-ecological zone were less than 100, indicating that the goats were intended for meat production. The results, however, were at odds with those of Assefa et al. [24], who found that PRI was greater than 100 in each of the study’s agro-ecological zones. The current study’s findings corroborated those of Dea et al. [28], who found that goats from Arbamminch-Zuria had PRI values below 100.

Thirdly, the study investigated how the agro-ecological zone affected the qualitative characteristics. The coat colour pattern, coat colour type, back profile, ear orientation, presence of horns, horn shape, and horn orientation were all examined in the study. According to the results of the current study on coat colour pattern, the four agro-ecological zones of the province of Limpopo had patchy dominating patterns. The results of the present investigation were not consistent with those of Zekele and Melese [29], who found that among native goats in the Gamo Gofa zone, the most common colour pattern was plain. The results of the current study also showed that the coat colour pattern of goats did not significantly differ amongst their population in any of the four agro-ecological zones. The results of this study differed with those of Tilahun et al. [30], who found that there was a plain prevailing pattern and a substantial variation in coat colour pattern across three districts. Similar results were obtained by Bekalu [31], which also indicated that the majority of goats in the West Gojam zone had patchy coloration. On the other hand, Takele [32] presented data that differed from those of the current study, reporting that the population of goats from three zones in Shabelle, South Eastern Ethiopia, was dominated by a plain colour pattern.

The results of the current study on coat colour type showed that, with the exception of the semi-arid zone, brown dominating colour was typical in three agro-ecological zones. The study showed that the population’s coat colour type was unaffected by the agro-ecological zone. According to research by Baldan et al. [33], Erdeneburen, Ulgii, and Bayan-Uul, respectively, have distinct dominant coat colour types, such as black, red, and mixed colours. Muluneh and Tadesse [34] revealed results that were comparable to those of the current study, showing that distinct colour types predominated in various zones. This might be due to the farmers preferences when selecting animals to breed. Arenas-Báez et al. [1], indicated that environmental conditions also drive the colour and pattern of the goats and the socio-cultural purposes. It was believed that darker animals easily adapt to the cold weathers as compared to the lighter ones and they do have important socio-cultural and economic values in the rural communities [35].

The current study showed that the back profile was dominated by the straight profile in three agro-ecological zones: semi-arid, sub-humid, and arid. The agro-ecological zones did not significantly affect the back profile. The current study results were not in agreement with those of Aseged et al. [36], who found that the predominant back profile in the goat population from to zones was a slope up towards the rump. According to the current study, every goat from each of the four agro-ecological zones had horns. Similar results were found in Ofori et al. [37], which found that all goats from four Ghanaian agro-ecological zones possessed horns. The results of the current study, which included goats from various Ghanaian zones, were different from those published by Zekele and Melese [29]. According to Aseged et al. [36], certain goats from different zones have polls in addition to horns. The presence of the horns assists this indigenous to protect themselves or fight with the predators in the veld and when the males had to fight one another for mating. The results of the current study on horn form showed that straight horns predominated in sub-humid areas, whereas curved horns prevailed in three of the province of Limpopo’s agro-ecological zones. The study also showed that the horn form did not significantly differ between the agro-ecological zones. Similar findings from the current study were reported by Seid et al. [38], who discovered that different horn shapes, straight horns from Amuru and Horro zones, and U-shaped horns from Guduru, predominated in different agro-ecological zones. Similar findings to the current study were published by Tilahun et al. [30], where the study’s common orientation was backward. Alubel [39], who noted that backward was the predominant orientation in the Central Highland and Abergelle goat populations, highlighted the findings even more. Additionally, according to Chokoe et al. [35], the predominant orientation in each of South Africa’s zones is backward horn. The results of the current investigation showed that semi-pendulous ear orientation was the dominant ear orientation. The findings of Muluneh et al. [40], were in contrast with the findings of the current study where horizontal orientation was dominating in three zone of West Gojjam zone. In addition, Whannou et al. [7] reported findings that differed from those of the current study, showing that erect ear orientation predominated in three Beninian agro-ecological zones.

## Conclusion

The study concludes that different agro-ecological zones in Limpopo Province of South Africa exhibit variance in the morphometric features of Nguni goats. Given that the region is notorious for being dry and hot, the farmers may have supplemented their goats throughout the year, leading to the high value of morphometric features in the goats from arid zones. The Nguni goats from four agro-ecological zones in Limpopo Province are considered light to medium meat goats, according to the structural indices. All Nguni goats had curved horn shapes followed by straight ones, and they were distinguished by their patchy coat colour pattern, brown dominating colour type, straight back profile, and slope up the rump. Backward horn orientation was the most common, followed by curved horn form and semi-pendulous ear orientation. The farmers’ preferences are reflected in the qualitative characteristics. The morphological characteristics of Nguni goats are unaffected by the agro-ecological zone, vegetation, or climate variations. The information from this study can be used to improve and conserve genetic resources of the Nguni goats. Furthermore, studies needed to be conducted on genetic characterization using genetic markers to combine the information from morphological variation of Nguni goats.

## Supporting information

S1 File Raw data of qualitative traits. It is in an Excel file with variables fully explained in column AI-AP on the sheet.
(XLSX)

S2 File Raw data of quantitative traits. It is in an Excel file with variables fully explained in column P, R, S and T of the sheet.
(XLSX)
